# Effects of metformin withdrawal after long and short term treatment in PCOS: observational longitudinal study

**DOI:** 10.1186/s13098-021-00660-5

**Published:** 2021-04-12

**Authors:** Nika Aleksandra Kravos, Andrej Janež, Katja Goričar, Vita Dolžan, Mojca Jensterle

**Affiliations:** 1 Department of Endocrinology, Diabetes and Metabolic Diseases, University Medical Centre Ljubljana, Zaloška 7, 1000 Ljubljana, Slovenia; 2 Faculty of Medicine, University of Ljubljana, Vrazov trg 2, 1000 Ljubljana, Slovenia; 3 Institute of Biochemistry, Faculty of Medicine, University of Ljubljana, Vrazov trg 2, 1000 Ljubljana, Slovenia

**Keywords:** Metformin, Legacy, PCOS, Obesity

## Abstract

**Background:**

Metformin plays a consolidated role in the management of polycystic ovary syndrome (PCOS). However, there is no clear answer on how long we should treat and on how long its beneficial impact sustain after we stop treatment. We compared the effects of metformin withdrawal after long-term (LT) and short term (ST) treatment in PCOS women that had previously well responded to metformin.

**Methods:**

We conducted observational longitudinal study including 44 PCOS women (31 (28–36) years and BMI 32.5 (27.7–34.9) kg/m^2^) that were followed for 6 months after metformin withdrawal. Prior inclusion, ST group had been treated with metformin on average for 1.03 ± 0.13 year, LT group for 5.07 ± 2.52 years. We followed anthropometric, metabolic, reproductive parameters and eating behavior as assessed by TFEQ-R18.

**Results:**

After metformin withdrawal, ST group gained significant amount of weight (from 92 (75.5–107.3) kg to 96 (76–116) kg; p = 0.019). Weight tended to increase also in LT users (from 87 (75–103) to 87 (73–105) kg; p = 0.058). More women in LT group maintained stable weight (27% in LT group vs 15% in ST group). Eating behavior deteriorated in both groups. Withdrawal of metformin resulted in a decrease of menstrual frequency (6 (6–6) to 6 (4–6) menstrual bleeds per 6 months; p = 0.027) and in borderline increase of androstenedione (6.4 (4.6–7.6) to 7.8 (4.8–9.6) nmol/L; p = 0.053) in LT group. Waist circumference, HOMA and glucose homeostasis remained stable in both groups. There were no differences between groups at 6-month follow up.

**Conclusion:**

Collectively, present study implies some metabolic and endocrine treatment legacy in both groups as well as some group-specific deteriorations in clinical parameters 6 months after metformin withdrawal.

*Trial registration*: The study is registered at Clinical Trials with reference No. NCT04566718

## Background

Metformin plays a consolidated role in management of polycystic ovary syndrome (PCOS) [1, 2]. It decreases hyperinsulinemia, hyperandrogenemia, improves menstrual cycles and pregnancy rates [3, 4]. Although these favorable effects are individually modest, they seem to be collectively sufficient to confer therapeutic benefits in metabolic and reproductive aspects of the syndrome [5]. By reducing cardiovascular risk factors [1, 2, 4–6], metformin also has a promising position in prevention of cardiometabolic sequelae of PCOS, rated as 1.43-fold increased risk of coronary arterial disease, cardiovascular disease, myocardial infarction, angina, heart failure, and ischemic heart disease in reproductive age PCOS patients [7].

Treatment with metformin is well established for patients with PCOS and impaired glucose homeostasis or type 2 diabetes mellitus (T2DM), when lifestyle intervention (LSI) is insufficient and for management of menstrual irregularity if women are unable to take oral contraceptives [1, 2, 8]. The latest international guidelines further advise a consideration of metformin as adjunct to LSI in adult women with PCOS with body mass index (BMI) ≥ 25 kg/m^2^, regardless of the presence of glucose disturbances and menstrual irregularity, for management of weight and preventing or slowing progression to adverse cardiometabolic outcomes [1].

The evidence supporting these recommendations and current clinical practice [1, 2] are mainly provided from short term studies and meta-analysis designed from 3 up to 12 months [9, 10]. Long-term studies are very limited [3, 9, 11, 12]. The longest prospective study lasted 4 years and reported initial weight reduction in the first year followed by stabilization of weight along 4 years, particularly in women with BMI over 25 kg/m^2^ [12]. At first year on metformin and diet, insulin resistance (IR) as assessed by homeostasis model assessment (HOMA-IR) was 33% lower than entry levels and remained the same for the following years [12]. Furthermore, long-term treatment with metformin-diet appers to be antiatherogenic by virtue of reductions of LDL-C and increments in HDL-C. The longest 10 year-retrospective study demonstrated that metformin treatment of overweight-obese women with PCOS resulted in reduction and stabilization of weight, improvements of menstrual function and androgen profile and in low conversion rate to diabetes [13]. However, less than 25% of patients remained adherent to metformin for more than 5 years with further dropout to only 6% of those still adherent to metformin at the 10th year of follow up [13].

Given the lack of long term data, it is not known whether metformin should be considered as a symptomatic or as a curative therapy. It is also not clear for how long metformin should be prescribed or whether there is any treatment legacy effects after its suspension.

## Aim of the study

We aimed to compare the consequences of metformin withdrawal after long as opposed to short term prior treatment in overweight/obese women with PCOS who had previously responded well to metformin by means of moderate weight loss, improved menstrual frequency and sustained normal glucose homeostasis.

## Methods

### Design and setting of the study

We conducted observational longitudinal cohort study including 44 overweight/obese PCOS women aged 31 (28–36) years and BMI 32.5 (27.7–34.9) kg/m^2^ that were followed for 6 months after metformin withdrawal. Inclusion criteria were PCOS defined by the Rotterdam criteria, phenotype A, characterized by concomitant presence of clinical or biochemical hyperandrogenism, ovulatory dysfunction and polycystic ovarian morphology (PCOM) [14, 15], continuous treatment with metformin monotherapy 1000 mg BID, age > 18 years, BMI ≥ 25 kg/m^2^, normal glucose homeostasis on metformin treatment prior the inclusion, and informed consent to discontinue metformin according to the study protocol. Adherence to metformin in preinclusion period was checked by patient’s self-report. Exclusion criteria were type 1 or T2DM, impaired glucose tolerance, weight gain more than 5% within 3 months prior the inclusion, arterial hypertension, pregnancy. Prior to inclusion, short-term (ST) group had been treated with metformin for 1 year (1.03 ± 0.13 years), long-term (LT) group continuously for at least 3 years (5.07 ± 2.52 years). The rationale behind selecting the treatment time in ST and LT groups was based on previous observations that the most meaningful effect of metformin has been demonstrated within first year of treatment, while during the following years of treatment the improved outcomes remained stable [13]. Out of 250 patients treated with metformin after a year at the outpatients Department of Endocrinology, Diabetes and Metabolic Diseases, University Medical Center Ljubljana, 77 were eligible for enrolment into ST group, 23 agreed to participate. Out of 400 patients, who had been treated with metformin for over 3 years, 38 were eligible for enrolment in the LT group, 21 agreed to participate, all of them were included. The study was ongoing between March and December 2019. After inclusion, all women discontinued metformin for 6 months. The stable LSI was advised throughout the observation period. Pregnancy was not specifically restricted.

### Experimental protocol

The entry data for the body weight, menstrual frequency and androgens at the beginning of metformin treatment were collected for all participants from the medical records.

At baseline and after 6 months of follow up, all patients underwent history check-up and standard anthropometric measurements: height, weight, waist circumference, blood pressure (BP). A fasting blood was drawn for determination of glucose, insulin, luteinizing hormone (LH), follicle-stimulating hormone (FSH), androstenedione, total and free testosterone followed by a standard 75 g oral glucose tolerance test (OGTT). At the entry point and at the end of the study they completed The Three-Factor Eating Questionnaire-R18 (TFEQ-R18) for assessment of eating behavior [16]. After 3 months of withdrawal from metformin all women came for a safety check-up visit to determine glucose homeostasis with standard OGTT.

### Anthropometric measurements

Height was measured with wall-mounted stadiometer to the nearest ± 1 cm. Body weight was measured with body weight scale to the nearest 1 kg. BMI was calculated as the weight in kilograms divided by square of height in meters. Waist circumference was measured in a standing position midway between the lower costal margin and the iliac crest.

### Assays

Glucose levels were determined using a standard glucose oxidase method (Beckman Coulter Glucose Analyzer, Beckman Coulter Inc CA, USA). Insulin was determined by solid-phase enzyme-labeled chemiluminescent immunometric assay (Immulite 2000 XPi System, Siemens Healthcare, United Kingdom). HOMA-IR score calculation was applied as a measure for IR. The estimate of IR by HOMA-IR score was calculated with the following formula: fasting serum insulin (mU/L) × fasting plasma glucose (mmol/L)/22.5 [17]. HOMA-IR score values 2.0 were considered as a cut-off point for IR as published previously [18]. Impaired glucose tolerance (IGT) was identified by 2 h glucose levels between 7.8 and 11.0 mmol/L, as defined by the American Diabetes Association criteria [19]. Androstenedione was measured by specific double antibody RIA using 125 I-labeled hormones (Diagnostic Systems Laboratories, Webster, Tx). Total and free testosterone levels were measured by coated tube RIA (DiaSorin, S. p. A, Salluggia, Italy and Diagnostic Products Corporation, LA, respectively). LH and FSH were measured using immunometric assay (Diagnostic Products Corporation, LA). Intra-assay coefficient of variation (CV) for androstenedione ranges from 5.0 to 7.5%, inter-assay CV from 4.1 to 11.3%; for free testosterone, intra-assay CV is 7.7–19.3% and inter-assay CV is 6.4–13.2%; for total testosterone, intra-assay CV is 5.1–16.3% and inter-assay CV is 7.2–24.3%. Intra-assay CV for SHBG is 2.5–5.3% and inter-assay CV is 4–6.6%.

### Determination of metabolic syndrome

According to the International Diabetes Federation definition, the metabolic syndrome in women is defined as central obesity (defined as waist circumference > 80 cm), plus any two of the following four factors: increased triglycerides ≥ 1.7 mmol/L, reduced HDL cholesterol < 1.29 mmol/L, increased BP (systolic BP ≥ 130 mmHg or diastolic BP ≥ 85 mmHg), increased fasting plasma glucose ≥ 5.6 mmol/L [19]. Since normal BP and fasting plasma glucose within normal reference range were inclusion criteria, participants were categorized as having metabolic syndrome based only on waist circumference and dyslipidemia.

### Menstrual frequency

Menstrual regularity was defined as number of bleeds per 6 months using self reported menstrual intervals based on a dairy review.

### Assessment of eating behavior

Eating behavior was assessed by using a Slovenian translation of Three-Factor Eating Questionnaire TFEQ-R18 [16]. The instrument is a shortened and revised version of the original 51-item TFEQ [20]. The translation of the Slovenian version had been back translated by a native English speaker and evaluated as required. The questionnaire measures three different aspects of eating behavior: cognitive restraint (CR) referring to conscious restriction of food intake in order to control body weight or to promote weight loss, uncontrolled eating (UE) referring to tendency to eat more than usual due to a loss of control over intake accompanied by subjective feelings of hunger, and emotional eating (EE) referring to inability to resist emotional cues. The TFEQ-R18 consists of 18 items on a 4-point response scale (definitely true/mostly true/mostly false/definitely false). Responses to each of the 18 items are given a score between 1 and 4 and item scores are summed up into scale scores for CR, UE, and EE [16, 20]. The raw scale scores are transformed to a 0–100 scale [raw score−lowest possible raw score)/possible raw score range × 100] and the commonly used ‘‘half-scale’’ method is utilized to compensate for missing data on some items. Higher scores in the respective scales are indicative of greater CR, UE, or EE.

### Statistical analysis

Continuous and categorical variables were represented with median with interquartile range (25–75%) or frequencies, respectively. Nonparametric Mann–Whitney was used to compare the distribution of continuous variables between different groups, while nonparametric Wilcoxon signed-rank test was used to compare continuous variables for related samples. Fisher’s exact test was used to compare categorical variables between different groups, while McNemar test was used to compare categorical variables for related samples. P values of < 0.05 were considered statistically significant. IBM SPSS Statistics, version 21.0 (IBM Corporation, Armonk, NY, USA) was used for all statistical analyses. Based on average weight increase after metformin withdrawal, we were able to detect differences in weight change of 3.6 kg with 80% power.

## Results

Four patients were lost due to drop out: two of them from ST group became pregnant, one from each group were excluded due to protocol violation. 40 women, 20 from ST aged 31 (28.3–35.5) years with BMI 32.7 (26.9–34.7) and 20 from LT group aged 32 (26–40) years with BMI 32.5 (27.7–38.8) finished the study according to the protocol.

### Pre-inclusion period

Prior the inclusion, the weight reduction was not significant in either group (women from ST group lost 3 kg from 95 (81.8–111.8) to 92 (75.5–107.3) kg; p = 0.147 in the 1st year of the metformin treatment; LT group lost 4 kg form 91 (79–100) to 87 (75–103) kg; p = 0.442) during the long term metformin treatment. There was no statistically significant difference between the groups in the response to metformin treatment regarding weight reduction. Metformin treatment increased menstrual bleeding from 4.5 to 6 menstrual bleedings per 6 months in ST group and from 3.5 up to 6 menstrual bleedings per 6 months in LT group (p = 0.041 and p = 0.003, respectively). Free testosterone and androstenedione decreased during ST metformin intervention (8.3 to 6.5 pmol/L; p = 0.007 and 7.1 to 5.4 pmol/L; p = 0.015 respectively). Total testosterone (2 to 0.95 nmol/L; p = 0.006) and androstenedione (6.9 to 6.4 nmol/L; p = 0.004) decrease during LT intervention prior the inclusion.

### Baseline

The two groups did not differ in any baseline parameters including age, body mass, BMI, waist circumference, blood pressure, number of menstrual bleeds per 6 months, parameters of TFEQ-18 (UE, EE, CR), plasma glucose and insulin level during OGTT and hormonal status (level of LH, FSH, androstenedione, total and free testosterone).

### Anthropometric measurement

6 months after metformin withdrawal women in ST group gained on average 4 kg (p = 0.019) (Table 2). Weight tended to increase also in LT group, but the increase was not statistically significant (from 87 (75–103) to 87 (73–105); p = 0.058) (Table 3). More women in LT group maintained stable weight (27% in LT group vs 15% in ST group). During follow up, three patients (one in ST group, two in LT group) complained over poor weight control. After they completed the study protocol, they immediately asked for re-introduction of metformin.

Waist circumference did not significantly increase in either group 6 months after metformin suspension (Tables 2 and 3). At the end of observation period there was no statistically significant difference in anthropometric parameters between the groups (Table 1).

**Table 1:** Comparison of characteristics of patients in short-term (ST) and long-term (LT) group 6 months after withdrawal

	ST group N = 20	LT group N = 20	p
Age (years)	31 (28.3–35.5)	32 (26–40)	0.770
Body weight (kg)	96 (76–116)	87 (73–105)	0.586
BMI (kg/m^2^)	33.3 (26.6–39.7)	32.5 (28.1–37.8)	0.964
Waist circumference (cm)	107 (89.5–118.5)	102 (90–113)	0.440
Systolic blood pressure (mmHg)	123 (113.5–137.5)	118 (111–122)	0.170
Diastolic blood pressure (mmHg)	84 (80–103.5)	85 (74–86)	0.142
No. of menstrual bleeding in last 6 month	6 (4.5–6)	6 (4–6)	0.586
TFEQ-R18: cognitive restraint (%)	61.1 (45.8–84.7)	47.2 (37.5–61.1)	0.085
TFEQ-R18: emotional eating (%)	22.2 (13.9–52.8)	33.3 (11.1–58.3)	0.631
TFEQ-R18: uncontrolled eating (%)	33.3 (10.2–40.7)	29.6 (18.5–42.6)	0.631
Total cholesterol (nmol/L)	4.6 (3.1–6.1)	4.9 (4.3–5.2)	0.786
HDL (mmol/L)	1.4 (1.1–1.7)	1.6 (1.2–1.7)	0.496
LDL (mmol/L)	2.1 (1.6–3.6)	2.5 (2.1–3.1)	0.339
TG (mmol/L)	1.4 (0.8–2)	1.3 (0.8–1.9)	0.964
FSH (IU/L)	5.2 (3.3–6.2)	5.4 (3.2–6.3)	0.683
LH (IU/L)	9.5 (5.3–16.2)	6.1 (2.8–8.4)	0.130
SHBG (nmol/L)	32.6 (20.4–75.7)	39.7 (32.5–56.5)	0.458
Total testosterone (nmol/L)	0.83 (0.69–1.5)	0.73 (0.69–1.36)	0.618
FAI score (%)	3 (1–4)	2 (2–3)	0.440
Free testosterone (pmol/L)	5.8 (4.9–7.2)	6.9 (4.8–7.5)	0.496
Androstenedione (nmol/L)	6.8 (4.6–9.9)	7.8 (4.8–9.6)	0.683
Glucose OGTT 0 (mmol/L)	4.9 (4.5–5.5)	5.3 (4.7–5.6)	0.316
Glucose OGTT 120 (mmol/L)	5.5 (4.2–7.3)	5.4 (4–6.5)	0.586
Insulin OGTT 0 (mU/L)	8.8 (2.3–21.9)	6.6 (2.6–12.9)	0.586
Insulin OGTT 120 (mU/L)	66.5 (37.2–174.5) [3]	45.7 (43.1–81.3)	0.170
HOMA-IR score	1.7 (0.5–4.9)	1.6 (0.5–2.7)	0.786

### Menstrual regularity

Menstrual frequency maintained stable in ST group (p = 0.715) and significantly decreased in LT group (p = 0.027) (Tables 2 and 3). There were no statistically significant difference between both groups at the end of the follow up period (Table 1). Five patients (2 from ST group and 3 from LT group) complained over irregular menstrual cycle. After they completed the study protocol, they immediately asked for metformin treatment.

**Table 2:** Comparison of characteristics of patients in short-term (ST) group at baseline and after 6 months of withdrawal

	Baseline	After 6 months	p
Body weight (kg)	92 (75.5–107.3) 93.9 ± 23.7	96 (76–116) 98 ± 25.3	**0.019**
BMI (kg/m^2^)	32.7 (26.9–34.7) 33.7 ± 9.5	33.3 (26.6–39.7) 35.2 ± 10.2	**0.023**
Waist circumference (cm)	106.5 (92–119.8)	107 (89.5–118.5)	0.722
Systolic blood pressure (mmHg)	124.5 (113.8–130)	123 (113.5–137.5)	0.688
Diastolic blood pressure (mmHg)	84.5 (80–98.5)	84 (80–103.5)	0.118
No. of menstrual bleeding in last 6 month	6 (4.3–6)	6 (4.5–6)	0.715
TFEQ-R18: cognitive restraint (%)	61.1 (50.0–66.7)	61.1 (45.8–84.7)	0.682
TFEQ-R18: emotional eating (%)	22.2 (0.0–77.8)	22.2 (13.9–52.8)	0.319
TFEQ-R18: uncontrolled eating (%)	22.2 (3.7–40.7)	33.3 (10.2–40.7)	**0.034**
Total cholesterol (nmol/L)	4.7 (3.3–5.5)	4.6 (3.1–6.1)	0.562
HDL (mmol/L)	1.4 (1.1–1.5)	1.4 (1.1–1.7)	0.474
LDL (mmol/L)	2.4 (1.6–3.5)	2.1 (1.6–3.6)	0.858
TG (mmol/L)	1.2 (0.8–1.7)	1.4 (0.8–2)	0.723
FSH (IU/L)	4.7 (2.9–6.4)	5.2 (3.3–6.2)	1.000
LH (IU/L)	7.7 (5.4–9.5)	9.5 (5.3–16.2)	0.328
SHBG (nmol/L)	31.8 (22–68.2)	32.6 (20.4–75.7)	0.552
Total testosterone (nmol/L)	1.02 (0.69–1.58)	0.83 (0.69–1.5)	0.110
FAI score (%)	3 (2–4)	3 (1–4)	0.473
Free testosterone (pmol/L)	6.5 (5.9–8.4)	5.8 (4.9–7.2)	0.382
Androstenedione (nmol/L)	5.4 (4.3–7)	6.8 (4.6–9.9)	0.075
Glucose OGTT 0 (mmol/L)	5.3 (4.9–5.6)	4.9 (4.5–5.5)	**0.021**
Glucose OGTT 120 (mmol/L)	5.8 (5.1–8.1)	5.5 (4.2–7.3)	0.506
Insulin OGTT 0 (mU/L)	0.7 (0.6–1.3)	0.8 (0.5–1.4)	0.239
Insulin OGTT 120 (mU/L)	69 (42.3–124.3)	66.5 (37.2–174.5)	0.249
HOMA-IR score	2.5 (1.4–4.9)	1.7 (0.5–4.9)	0.221

**Table 3:** Comparison of characteristics of patients in long-term (LT) group at baseline and after 6 months of withdrawal

	Baseline	After 6 months	p
Body weight (kg)	87 (75–103) 89.4 ± 15.8	87 (73–105) 91 ± 17.1	0.058
BMI (kg/m^2^)	32.5 (27.7–38.8) 32.8 ± 5.7	32.5 (28.1–37.8) 33.4 ± 6.2	0.056
Waist circumference (cm)	98 (92–110)	102 (90–113)	0.501
Systolic blood pressure (mmHg)	118 (113–130)	118 (111–122)	0.211
Diastolic blood pressure (mmHg)	87 (81–92)	85 (74–86)	**0.010**
No. of menstrual bleeding in last 6 month	6 (6–6)	6 (4–6)	**0.027**
TFEQ-R18: cognitive restraint (%)	66.7 (61.1–83.3)	47.2 (37.5–61.1)	**0.003**
TFEQ-R18: emotional eating (%)	33.3 (11.1–55.6)	33.3 (11.1–58.3)	0.522
TFEQ-R18: uncontrolled eating (%)	22.2 (7.4–44.4)	29.6 (18.5–42.6)	0.469
Total cholesterol (nmol/L)	4.9 (4.3–5.3)	4.9 (4.3–5.2)	0.733
HDL (mmol/L)	1.5 (1.2–1.8)	1.6 (1.2–1.7)	0.658
LDL (mmol/L)	2.6 (2.3–3.2)	2.5 (2.1–3.1)	0.454
TG (mmol/L)	1.3 (0.8–2.1)	1.3 (0.8–1.9)	0.874
FSH (IU/L)	5.4 (3.9–7)	5.4 (3.2–6.3)	0.977
LH (IU/L)	7.3 (2.7–11.1)	6.1 (2.8–8.4)	0.910
SHBG (nmol/L)	42.2 (28.5–76.4)	39.7 (32.5–56.5)	0.925
Total testosterone (nmol/L)	0.95 (0.69–1.1)	0.73 (0.69–1.36)	0.530
FAI score (%)	2 (1–4)	2 (2–3)	0.752
Free testosterone (pmol/L)	6.7 (4.4–8.4)	6.9 (4.8–7.5)	0.820
Androstenedione (nmol/L)	6.4 (4.6–7.6)	7.8 (4.8–9.6)	0.053
Glucose OGTT 0 (mmol/L)	5.1 (4.9–5.5)	5.3 (4.7–5.6)	0.800
Glucose OGTT 120 (mmol/L)	5.9 (4.6–6.9)	5.4 (4–6.5)	0.233
Insulin OGTT 0 (mU/L)	0.7 (0.5–1)	0.6 (0.4–0.8)	0.319
Insulin OGTT 120 (mU/L)	40.7 (13.2–80.9) [	45.7 (43.1–81.3)	0.826
HOMA-IR score	1.4 (0.6–2.1)	1.6 (0.5–2.7)	0.875

Legend of abbreviations used in every table: BMI: body mass index; TFEQ-R18: three factor eating questionnaire; HDL: high-density lipoprotein; LDL: low-density lipoprotein; TG: triglyceride; FSH: follicle stimulating hormone; LH: luteinizing hormone; SHBG: sex hormone-binding globulin

Values of androgens in the healthy matched population: total testosterone 0.3–3.5 nmol/L; free testosterone 0.14–7.0 pmol/L; SHBG: 18–114 nmol/L; androstenedione: 0.7–10.8 nmol/L; FAI score: free androgen index 0.3–4.4%; HOMA-IR: homeostasis model assessment of insulin resistance

### Metabolic changes

At 3-month safety check-up, none of the participants developed diabetes mellitus, therefore all patients continued with the study. After 6 months, none of them developed IGT or impaired basal glycemia or diabetes mellitus. There was also no increase in HOMA-IR (2.5 (1.4–4.9) to 1.7 (0.5–4.9), p = 0.221 in ST group and 1.4 (0.6–2.1) to 1.6 (0.5–2.7), p = 0.875 in LT group) and no increase in mean values of glucose and insulin during OGTT in either group (Tables 2 and 3).

At the beginning of the study, four women (20%) in ST group and five (25%) women in LT group had metabolic syndrome. After 6 months, additionally one woman in ST group and two women in LT group developed metabolic syndrome due to increase of serum triglyceride. There were no significant between arm differences (p = 1.0).

### Endocrine changes

Values of assessed hormones remained stable over the study period in both groups with borderline increase of androstenedione in LT group (6.4 (4.6–7.6) to 7.8 (4.8–9.6) nmol/L, p = 0.053) (Tables 2 and 3). 19 out of 20 (95%) of women in ST group and 15 out of 20 (75%) in LT group had biochemical characteristics of hyperandrogenemia (higher total testosterone and/or FAI, free testosterone, androstenedione) at baseline and 20 out of 20 (100%) of women in ST group and 15 out of 20 (75%) in LT group at the end of observational period after 6 months.

### Assessment of eating behavior

After cessation of metformin UE in the ST group increased from 22.2 (3.7–40.7) to 33.3 (10.2–40.7) (p = 0.034). In LT group CR decreased from 66.7 (61.1–83.3) to 47.2 (37.5–61.1) (p = 0.003). There were no significant changes in other measured aspects of eating behavior or between ST and LT groups (Tables 1, 2 and 3).

## Discussion

In ST users, withdrawal of metformin in obese PCOS resulted in regain of body weight and changed eating behavior. In LT users withdrawal resulted in changed eating behavior and menstrual irregularity. By contrast, waist circumference, IR, glucose homeostasis and the androgen profile, except borderline increase in androstenedione in LT group, remained stable implying some metabolic and endocrine treatment legacy of metformin in 6 month follow up. To the best of our knowledge, this is the first study that compared consequences of metformin withdrawal on clinical, metabolic and endocrine parameters in ST and LT prior users.

In the Diabetes Prevention Program Research Group (DPPRG) participants at high risk for T2DM that had been treated with metformin lost on average 2.1 kg during 2 years follow up [6]. The amount of weight change was associated with adherence, with highly adherent patients experiencing 3.5% reduction in body mass compared to neutral status in low adherent patients [6]. DPPRG also showed that metformin- related weight loss sustained over 9 years [6]. Similar results were retrospectively observed also in PCOS cohort followed for 10 years, with the greatest 3.7% weight reduction after the first year of metformin treatment and sustainable effect up to 10 years in participants that remained adherent to the treatment [13]. However, the real-word adherence and persistence with long term metformin treatment in women with PCOS is very low. The longest retrospective analysis of real-life cohort reported that the drop-out rates increased from about 10% in the first year, 35% in the second year, 50% in the third year, 65% in the fourth and up to 80% in the fifth year of metformin treatment [13]. Although poor compliance with LT treatment with metformin in PCOS is typically observed in everyday clinical practice [21], the consequences of withdrawal still remains unexplored.

We demonstrated that 6 months after metformin cessation women had significant weight regain, in particular in ST group. Weight tended to increase also in LT group, yet to the lesser extent and in lesser percentage of women, implying some longer lasting legacy of metformin in LT users. However, despite the observed weight gain, withdrawal from metformin did not resulted in increased waist circumference in either group. Mechanistically, metformin might counteract adipose tissue expansion through direct inhibition of adipogenesis with the specific role of metformin on visceral fat mass as opposed to subcutaneous fat compartments [5].

When analyzing eating behavior, we observed that after cessation of metformin women in ST group had increase in UE score. It is known that energy-dense foods, such as fat, were positively associated with UE scores [16], which means poorer control of food intake that was also reported by patients. In LT group we observed non-beneficial decrease in CR score. It is known that the higher CR scores as opposed to lower CR scores might be related to a greater propensity for dieting [16] and that low restrainers had reduced preference for “healthy” food, such as fish, vegetables, and fat-reduced food [16]. EE score that is usually associated with higher consumption of snack foods [16] remained stable in both groups.

In line with the observed changes in eating behaviour after metformin withdrawal in our study are the findings reporting that metformin in PCOS tends to restore central hormonal appetite regulators, in particular NPY-ghrelin axis [22]. Lactate-mediated, mild, metabolic acidosis may also drive some metformin-mediated appetite suppression [23]. In addition, metformin may affect appetite through the gut-brain axis via increased secretion of the weight-loss promoting incretin glucagon like peptide 1 and the anorectic hormone peptide YY [23].

Prior the inclusion, metformin restored menstrual cycles in both groups of our cohort. The well-established effectiveness of metformin in achieving normal menses is linked to several pathogenic mechanisms such as reducing IR and secondary hyperinsulinemia, indirect effect on the ovary and a direct effect on the endometrium [8]. Six months after withdrawal, we observed a gradual reduction in period cyclicity in LT group. ST group continued to have regular menstrual cycles post metformin withdrawal. Two of our patients from ST group conceived spontaneously without ovulation induction during observation period.

Furthermore, we observed that there was no significant change in the glucose, insulin or IR after discontinuation of the metformin treatment within 6 months follow up period. After 6 months, none of the participants developed IGT or impaired basal glycemia or T2DM. As opposed to our observations, study by Palomba et al. reported that 6 months after discontinuing metformin, there was a statistically significant increase in IR in normal weight anovulatory PCOS women who had previously received metformin of 1700 mg BID for 12 months, as compared with placebo and healthy subjects [11]. Notably, participants in the present study may not represent the usual obese women with PCOS since hypertension, T2DM and impaired glucose tolerance were exclusion criteria, and the immanent cardio-metabolic risk was lower than in a more typical population of women with PCOS and obesity.

Prior the inclusion, androgens decreased in both arms. After metformin withdrawal, we observed borderline increase of androstenedione in LT group and no change in other endocrine profile. The beneficial impact of metformin treatment on androgen profile could be explained by its indirect effect via the decrease in IR as insulin was shown to directly stimulate several steroidogenic enzymes in the ovary [24]. In addition, it has been demonstrated that metformin at therapeutic concentrations was able to directly suppress androstenedione production in human ovarian theca-like tumor cells in culture cells [5].

The main limitation of our study is relatively small number of patients that were included in each arm. However, the inclusion and exclusion criteria were highly selective and as seen from the described selection process, the majority of patients form our base did not fulfill them. Secondly, the duration of follow up is short and further changes in endocrine and metabolic parameters are expected over time in chronic metabolic disease like PCOS. The longer period of follow up was considered unethical since all women had been categorized as a phenotype with increased metabolic risk, in which metformin should be considered in clinical practice [1]. In most of the participants, a deterioration of at least one parameter was observed within 6 months after withdrawal. Five women asked for metformin re-introduction immediately after they completed the study protocol. 50% of participants opted to started with metformin after the study end and another 9 within the following 6 months.

The main strength of this study is that it assessed consequences on metabolic and endocrine parameters of metformin withdrawal comparing ST and LT users that had previously well responded to metformin by virtue of moderate weight loss, improved menstrual frequency and sustained normal glucose homeostasis prior the inclusion. Moreover, the two groups were comparable by all baseline criteria, including age. This is important, since ageing has impact on the natural progress of the disease and the potential baseline age differences between groups would further mask the assessment of legacy effect.

## Conclusions

In conclusion, we found that metformin discontinuation after 6 months of follow up resulted in regain of body weight and changed eating behavior in women with short-term metformin treatment and in changed eating behavior, menstrual irregularity and increase of androstenedione of borderline significance in women with LT metformin treatment. On the other hand, there seems to be some treatment legacy in ST and LT users in regard with sustainable IR, glucose homeostasis and overall androgen profile during 6 months follow up. Collectively, present study implies some metabolic and endocrine short term treatment legacy in both groups as well as some group specific deteriorations.

Due to small sample size and the lack of data regarding adherence to lifestyle interventions might have influenced on the results, further studies with larger sample size are needed to clarify these questions. There is still no clear answer on how long we should treat PCOS with metformin but it seems that when the patients with PCOS are overweight or obese, a continued treatment is needed for ongoing overall clinical benefit. The consequences of potential intermittent drug holidays in long term users should be further explored. Larger and longer lasting longitudinal studies are needed to tailor the benefit/risk profile on an individual patient basis. More data from such studies would also help to improve a very low adherence to long term metformin treatment in this population.

Other therapeutic alternatives for weight loss and restoration of menstrual cyclicity such as multicomponent lifestyle intervention including diet, exercise and behavioral strategies [25], myo-inositol [26] and GLP-1 based therapies [27, 28] also need further exploration in overweight/obese patients with PCOS and high metabolic risk who are metformin non-adherent or metformin-intolerant.

## Data Availability

The datasets used and/or analyzed during the current study are available from the corresponding author upon reasonable request.

## Abbreviations

PCOS: Polycystic ovary syndrome
LT: Long-term
ST: Short term
TFEQ-R18: The Three-Factor Eating Questionnaire-R18
HOMA: Homeostasis model assessment
IR: Insulin resistance
T2DM: Type 2 diabetes mellitus
LSI: Lifestyle intervention
BMI: Body mass index
BP: Blood pressure
LH: Luteinizing hormone
FSH: Follicle-stimulating hormone
OGTT: Oral glucose tolerance test
CR: Cognitive restraint
UE: Uncontrolled eating
EE: Emotional eating
IGT: Impaired glucose tolerance
DPPRG: Diabetes Prevention Program Research Group
